# A protocol for evaluating robustness to H&E staining variation in computational pathology models

**DOI:** 10.1016/j.jpi.2026.100702

**Published:** 2026-08-01

**Authors:** Lydia A. Schoenpflug, Nikki J. van den Berg, Sonali Andani, Nanda Horeweg, Jurriaan Barkey Wolf, Tjalling Bosse, Viktor Koelzera, Maxime W. Lafarge

**Affiliations:** a Department of Biomedical Engineering, University of Basel, Allschwil, Switzerland; b Institute of Medical Genetics and Pathology, University Hospital Basel, Basel, Switzerland; c Department of Pathology, Leiden University Medical Center, Leiden, the Netherlands; d Department of Radiation Oncology, Leiden University Medical Center, Leiden, the Netherlands

**Keywords:** Computational pathology, H&E staining, Domain generalization, Quality control, Model robustness

## Abstract

Sensitivity to staining variation remains a major barrier to deploying computational pathology (CPath) models as hematoxylin and eosin (H&E) staining varies across labs, requiring systematic assessment of how this variability affects model prediction. Yet, current evaluation practices rarely attribute performance changes to specific, measurable staining properties, limiting interpretable robustness assessment under controlled conditions. We introduce a three-step protocol that explicitly simulates H&E staining variation toward known, measurable targets, supporting controlled assessment of model performance shifts. Step 1: Select reference staining conditions, Step 2: Characterize test set staining properties, Step 3: Apply CPath model(s) under simulated reference staining conditions. Here, we construct a new reference staining library based on the PLISM dataset. As a use case, we evaluate 306 microsatellite instability (MSI) classification models on the SurGen colorectal cancer dataset (*n* = 738), including 300 attention-based multiple instance learning models trained on TCGA-COAD/READ across three feature extractors (UNI2-h, H-Optimus-1, and Virchow2), and six public models. Classification performance was measured as AUC, and robustness as the min-max AUC range across four simulated staining conditions (low/high H&E intensity, low/high H&E color similarity). Across models and staining conditions, classification performance ranged from AUC 0.769–0.911 (Δ = 0.142). Robustness ranged from 0.007 to 0.079 (Δ = 0.072), and showed a weak inverse correlation with classification performance (Pearson *r* = −0.28, 95% CI [−0.39, −0.17]). Thus, using MSI classification in colorectal cancer as a use case, we demonstrate that the proposed evaluation protocol enables robustness-informed CPath model selection and provides insight into how H&E staining conditions affect model performance, with the potential to support reliable deployment across clinical settings. Code and reference library are publicly available at https://huggingface.co/datasets/CTPLab-DBE-UniBas/staining-robustness-evaluation and https://github.com/CTPLab/staining-robustness-evaluation.

## Highlights

• Evaluation protocol quantifies CPath model robustness to H&E staining variation.
• Built a reference library of H&E stain properties from the PLISM dataset.
• 306 CRC MSI classification models evaluated under four H&E staining conditions.
• Robustness to staining variation is a key criterion for CPath model selection.

## Graphical abstract

**Figure:**
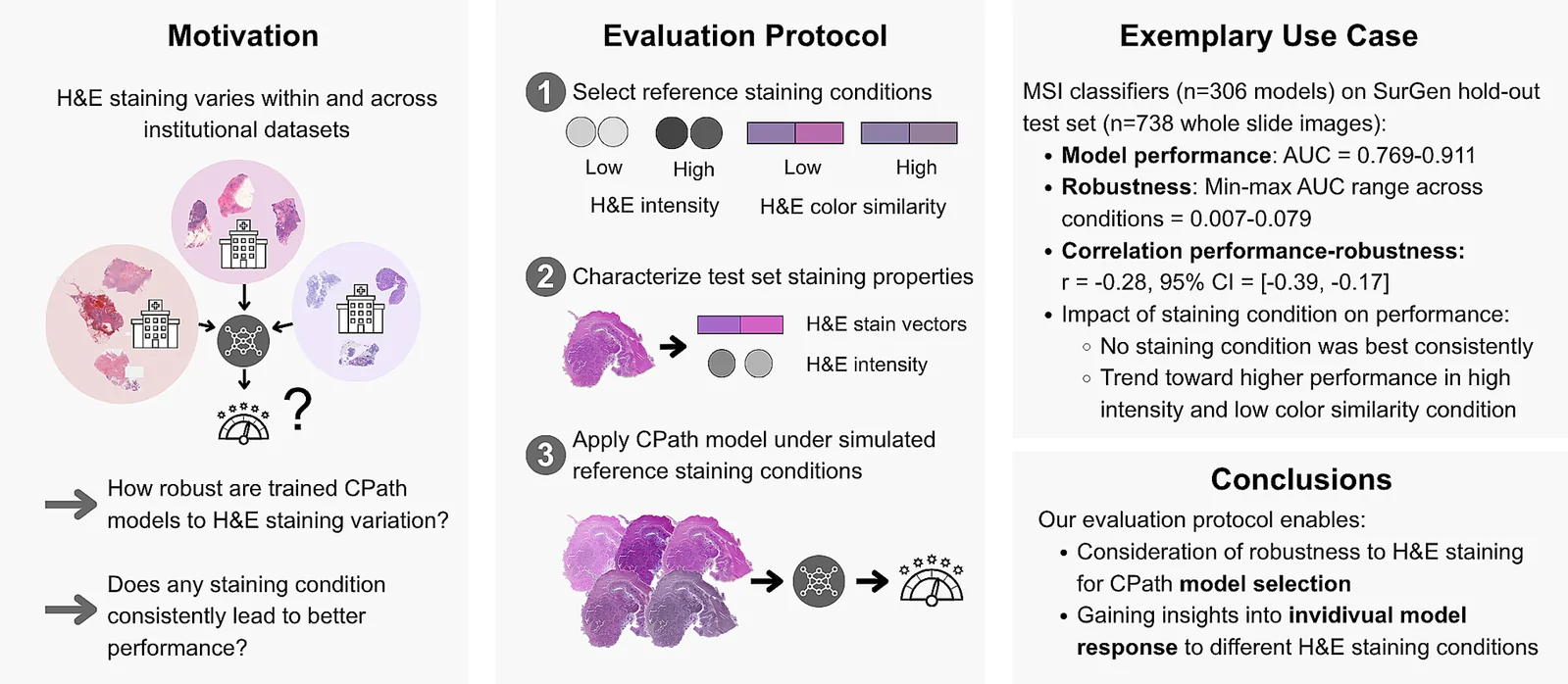

## Introduction

Hematoxylin and eosin (H&E) staining is central to diagnostic histopathology and forms the input of most computational pathology (CPath) pipelines. However, the appearance of whole-slide images (WSIs) of H&E-stained specimens exhibits substantial variability within and across labs due to differences in staining protocols, reagent concentrations, batch effects, scanner characteristics, and tissue processing workflows^1^. These differences produce shifts in stain intensity and color, and can alter morphological information, which can affect model performance when CPath models are applied to WSIs of specimens prepared at different timepoints or institutions^2^, ^3^, ^4^, ^5^, ^6^.

Prior work addressing staining-related domain shift can be broadly grouped into development-focused and evaluation-focused approaches. Development-focused methods, including stain normalization^7^, ^8^, ^9^, ^10^, stain augmentation^6^, ^11^, ^12^, ^13^, ^14^, ^15^, and domain-adversarial training^16^, ^17^, all aim to improve robustness during training. However, a critical gap arises in the context of modern CPath pipelines: existing methods primarily focus on improving encoder-level training, assuming encoders are trained locally. At the same time, the field has shifted toward large pre-trained foundation models, whose training is restricted to institutions with access to massive datasets and computational resources (e.g., UNI: >100 k WSIs, 32 GPUs^18^). As a result, most researchers no longer modify the encoder but instead train lightweight attention-based multiple instance learning (ABMIL) classifiers on top of features extracted by frozen foundation models, further referred to as two-stage training pipelines. In this setting, training-time strategies such as stain augmentation or normalization have reduced influence^19^, as their impact is constrained by the pre-trained feature representations. At the same time, current foundation models are not fully invariant to domain shifts, including staining variability: extracted features retain institution-specific information^20^, ^21^, ^22^, ^23^. Yet, pipelines based on foundation models generally outperform previous approaches, suggesting that adequate pretraining confers a measurable improvement in robustness to staining variation and reduces, but does not eliminate, sensitivity to H&E variability. Evaluation-focused studies have demonstrated that staining variability can affect performance more strongly than many common artifacts^24^, ^25^, ^26^, ^27^. Yet, existing evaluation strategies often rely on image-based references^25^, ^26^, generative transformations^28^, fixed multiplier perturbations^27^, or re-staining/re-scanning protocols^29^, ^30^, ^31^, which make it difficult to attribute performance changes to specific, quantifiable staining properties or to situate perturbations within a defined staining reference space. Taken together, these observations highlight a need for systematic evaluation methods that quantify the robustness to staining variation of trained two-stage CPath pipelines at inference time, and anchor perturbations to defined staining properties. Such methods can inform both model selection and diagnostic slide preparation, supporting data-driven quality control.

**Fig. 1:**
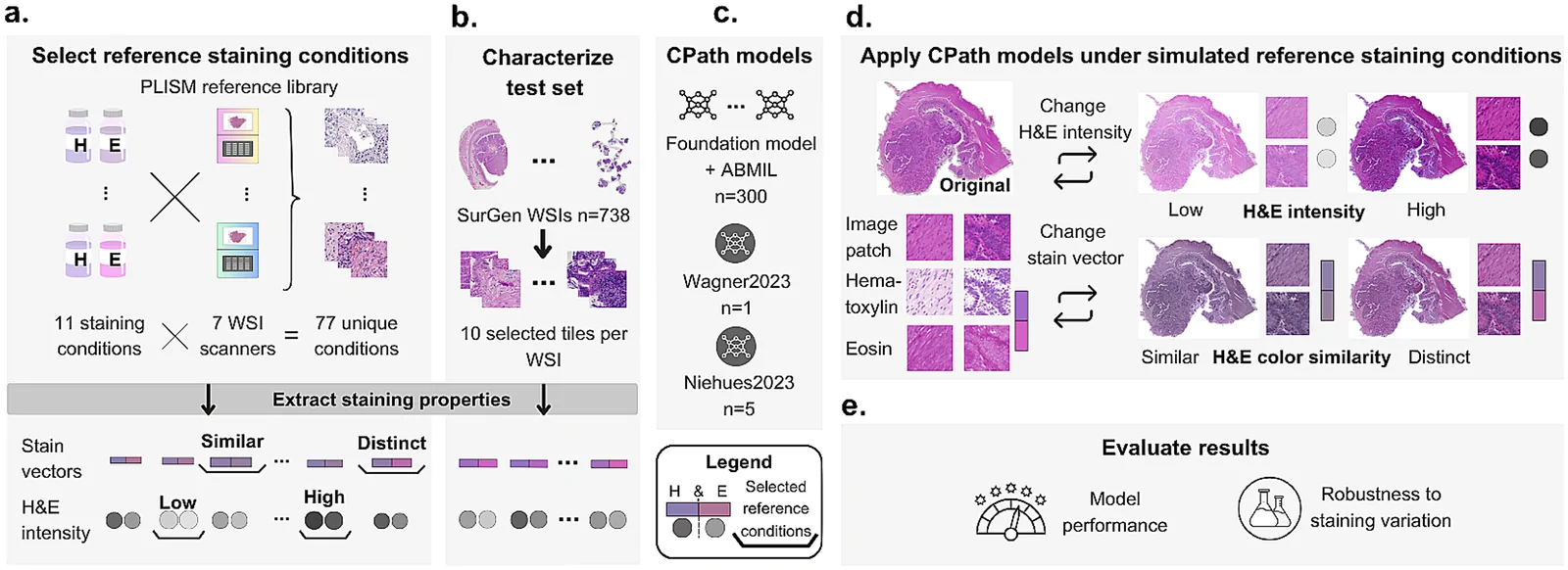
A protocol for evaluating robustness to staining variation of CPath models. (a) Select reference staining conditions from the PLISM reference library, representing distinct target H&E stain vectors and intensities. (b) Characterize the test set by estimating the staining properties of each SurGen slide using Macenko stain decomposition^9^. (c) State-of-the art MSI classification models, with *n* = 300 models reflecting plausible state-of-the-art models, which were trained on TCGA COAD/READ using one of three foundation models as feature extractors (Uni2-h, HOptimus1, and Virchow2) and ABMIL for aggregation. We also considered *n* = 6 publicly available models. (d) Apply MSI classification models under four simulated reference staining conditions by recomposing each SurGen slide using its estimated source stain vectors and intensities and the stain vectors and intensities of each selected PLISM reference condition, thereby generating four simulated versions of the original test set while preserving tissue morphology. (e) Evaluation of results by measuring model performance and robustness to staining variation.

To address this deficit, we introduce a protocol for systematically evaluating the robustness of CPath models under realistic H&E staining variations (Fig. 1). We construct a reference staining library from the Pathology Images of Scanners and Mobilephones (PLISM) dataset, characterizing the staining properties of tissue specimens prepared under 11 H&E staining conditions and digitized with seven WSI scanners. This library supports simulation of plausible staining variations while preserving tissue structure, thereby enabling quantitative assessment of model robustness. We demonstrate the protocol using MSI classification in colorectal cancer (CRC) as a use case, evaluating the robustness of 306 state-of-the-art models to staining variation on the publicly available SurGen cohort of 738 CRC patients. Overall, the protocol enables systematic, quantitative evaluation of staining-induced variability, and supports more reliable model deployment in clinical workflows.

## Methods

### Stain decomposition and controlled recomposition framework

We modeled the RGB pixel intensities of a histology image $I\in {\left[0,255\right]}^{H\times W\times 3}$ as a function of the optical density (OD) using the Beer-Lambert law

(1) $$\mathrm{OD}=-\log\left(\frac{I}{I_{0}}\right),$$

where $I_{0}$ denotes the illumination constant (typically 255). Following Macenko et al.^9^, the OD of each pixel is assumed to lie in the subspace spanned by the H&E stain vectors $s_{H},s_{E}\in R^{3}$. We further include a third residual vector $s_{R}$ orthogonal to the H&E plane to account for residual variation. Using this framework, each pixel's OD is represented as:

(2) $$\mathrm{OD}\approx i_{H}s_{H}+i_{E}s_{E}+i_{R}s_{R},$$

where $i_{H},i_{E},i_{R}\geq 0$ denote the stain intensities, quantifying the per-pixel staining magnitude. For image-level quantification, the H&E stain intensity is defined as the 95th percentile across all pixels. To robustly identify the H&E stain vectors, the OD matrix of filtered pixels is analyzed via singular value decomposition. The two dominant singular vectors define the H&E plane, in which the stain vectors are subsequently identified, and the third singular vector defines the residual vector. Pixels with extreme OD values are excluded to avoid artifacts. For staining simulations, OD projections are scaled to target H&E intensities and recombined using the target stain vectors. In pixels where the residual component is dominant (residual OD value >0.1), indicating that the identified H&E stain vectors are insufficient to capture the pixel color, its contribution is reduced by an empirically determined factor of 0.01 to avoid unrealistic color shifts upon reconstruction.

### Datasets

#### PLISM

The PLISM dataset^32^ comprises aligned image patches from 46 human tissue types, stained under 13 H&E staining conditions and digitized using 13 imaging devices, including 7 WSI scanners, with recorded slide preparation parameters^e^. This design enables extraction of realistic H&E staining intensities and stain vectors for downstream simulation. As this study is focused on the analysis of WSIs, we restricted the analysis to the subset of images patches of the PLISM dataset captured with WSI scanners (PLISM-wsi). We further excluded the HR and GIV staining conditions for our study, as we consider the overnight hematoxylin exposure to produce an unrealistically intense staining, resulting in 11 considered staining conditions. The final subset includes 263,109 patches, corresponding to 3417 aligned patches across 77 unique stain-device combinations (Supplementary Tables A.1 and A.2).

#### TCGA COAD/READ

The TCGA Colon and Rectum Adenocarcinoma cohorts (COAD, READ) were used as the development set for training and validating a set of plausible “black-box” MSI classifiers. A total of 625 diagnostic WSIs of H&E-stained formalin-fixed paraffin-embedded (FFPE) resection specimens from 616 patients were downloaded from the Genomic Data Commons portal^f^ of which 25 WSIs were excluded due to file corruption, yielding 600 WSIs from 591 patients. MSI status was obtained for all patients from two sources available through cBioPortal^g^: (i) the PanCancer Atlas^33^, providing next-generation sequencing (NGS)-derived MSI calls using MANTIS^34^, and (ii) the original TCGA publications^35^, which reported PCR-based MSI status. When both labels were available, NGS-derived MSI calls were prioritized to ensure a consistent, genome-wide reference standard across the cohort.

#### SurGen

The SurGen dataset^36^ is a publicly available^h^ collection of WSIs of FFPE H&*E*-stained CRC resection and biopsy specimens and was used solely as a holdout test set in this study. It comprises 1020 WSIs from 843 patients and is divided into two subsets: SR386, containing primary colorectal tumors with matched genomic and clinical annotations, and SR1482, which includes both primary and metastatic cases. MSI status was derived from either PCR-based fragment analysis or mismatch-repair (MMR) immunohistochemistry (IHC), with PCR-based calls preferred where available. For IHC-derived labels, loss of expression of one or more MMR proteins was interpreted as microsatellite instable, intact expression of all MMR proteins was interpreted as microsatellite stable. Patients with WSIs exclusively from non-colorectal metastatic sites (*n* = 92), missing MSI status (*n* = 11), or providing insufficient tissue (*n* = 2) were excluded, resulting in 738 patients with 892 WSIs. When multiple slides were available for the same patient, the first available WSI was selected.

### Models

#### Simulated models

To reflect the diversity of models that may realistically be developed or received by clinical implementers, we simulated a set of ABMIL models trained on TCGA COAD/READ using foundation-model-based pipelines. Tissue detection was performed on all WSIs using the tissue segmentation model of Bándi et al.^37^, followed by tiling into 224px × 224px patches at 1 μm/px for feature extraction. No stain augmentation or normalization was applied, following established ABMIL training frameworks^19^. To induce realistic variability, we sampled across multiple hyperparameters: three foundation model feature extractors (UNI2-h^i^^,^^38^, H-Optimus-1^j^^,^^39^, Virchow2^k^^,^^40^) with 100 models per extractor, arbitrary random initialization seeds, two weight decay values (0 or $10^{-4}$), MSI-stratified 70:30 patient-level train-validation splits with or without institutional overlap, and random exclusion of 0–10 institutions per model while retaining at least 90% of the development dataset. This approach simulates variability in data splits, training methodology, and institutional coverage. Slide-level aggregation of patch-level features was performed using a gated ABMIL model adapted from Lu et al.^41^, with an additional pre-compression fully connected layer. Models were trained using AdamW^42^ with learning rate $5\cdot 10^{-5}$, momentum 0.95/0.999, cosine annealing, batch size 1, early stopping with five-epoch patience based on validation AUC, and a maximum of 30 epochs.

#### Public models

In addition to the simulated models, we included six publicly available, trained MSI classification models from two publications. The first Wagner et al.^43^, uses the CTransPath^44^ model for feature extraction from 512px × 512px image patches at 0.5μm/px (resized to 224px × 224px, 1.14 μm/px), followed by a transformer-based MIL (TransMIL) aggregator. The TransMIL model was trained on WSIs from over 13,000 CRC patients across 16 cohorts. The five other models^45^, use the RetCCL^46^ model for feature extraction with identical input format as CTransPath (224px × 224px, 1.14 μm/px) and gated ABMIL aggregation. These five models were trained with different dataset splits under a 5-fold cross-validation framework. We further refer to the public models based on their feature extractor and MIL aggregator combination as CTransPath+Wagner2023 and RetCCL+Niehues2023. Feature extractors and pretrained MIL weights were obtained from the authors' publicly released repositories^l^. Inference was performed without stain normalization and consisted of feature extraction using CTransPath or RetCCL, followed by aggregation with the respective pretrained MIL models to obtain slide-level MSI classifications.

### Protocol for evaluating robustness to H&E staining variation

Our protocol for evaluation of CPath model robustness to H&E staining variation comprises three stages. First, reference staining conditions are selected based on a reference library. Here, we first created a new reference library from the PLISM dataset. Second, the test set staining properties are characterized, specifically slide-wise stain vectors and intensities are extracted. Third, inference of the CPath model is performed under a controlled set of simulated staining conditions. Here, we present the application of this protocol to MSI classification models, evaluated and compared using the holdout SurGen test set.

#### Step 1: Select reference staining conditions

To enable reference selection we first created a new reference library from the PLISM dataset. Only tiles meeting quality criteria (Supplementary Table B.1) were included. Stain vectors and intensities were extracted for these tiles as described in Section Stain decomposition and controlled recomposition framework, and median values across tiles were computed for each stain-device combination to define representative H&E stain vectors and intensities. Stain intensity was quantified as the 95th percentile of H&E intensities in the stain-separated projections, capturing well-stained tissue while avoiding extreme artifacts (e.g., blood). For simulating intensity-based changes, the conditions with lowest and highest H&E intensity were selected. To simulate changes of H&E stain colors, we selected stain vector references reflecting common diagnostic hematoxylin formulations: Harris for the most distinct and Gill for the most similar H&E stain colors, where similarity was measured as the angle between H&E vectors in OD space. These references enable realistic simulation of both intensity- and color-based variations in H&E staining. Exemplary tiles and staining details for each reference can be found in Supplementary Fig. B.1 and Table B.2. All the stain vectors and intensities derived from the PLISM dataset were released as a public reference library^m^.

#### Step 2: Characterize test set staining properties

For each slide of the SurGen dataset, slide-level stain vectors and H&E intensities were obtained by sampling 10 tiles (448px × 448px at 0.5 μm/px) per slide that passed the tile quality criteria (Supplementary Table B.1). In rare cases where artifact colors affected decomposition (e.g. pink, orange, brown, or dark-red blood), quality criteria were manually adjusted until visual review of the derived stain vectors showed alignment with WSI stain appearance. Stain vectors and H&E intensity values were extracted as described for the PLISM reference conditions, and slide-level representatives were computed as the median across the sampled tiles. Slide-wise stain intensities and vectors then served as the basis for staining simulations during inference. To empirically assess the stability of slide-level stain property estimation from 10 tiles, we conducted a bootstrapping sensitivity analysis (Supplementary Material C). Subsets of *n* = 10 tiles were repeatedly drawn (1000 iterations) from a larger pool of *n* = 50 random tiles that passed quality control per WSI and the estimated stain properties were compared to the 50-tile reference to quantify sampling stability. All the slide-level stain vectors and intensities were made publicly available^n^.

#### Step 3: Apply CPath model under simulated reference staining conditions

Inference under controlled, simulated staining conditions on SurGen WSIs consisted of tissue detection with the tissue segmentation model of Bándi et al.^37^, followed by tiling into patches, matching the input size and resolution of the respective feature extractor as specified in the Models Section. Tiles are then decomposed into H&E channels using the slides' stain vectors, and are further recomposed based on the target PLISM references to simulate the target staining conditions as described in Section Stain decomposition and controlled recomposition framework. Experiments covered four PLISM-defined conditions: low vs. high H&E intensity and high vs. low H&E color similarity. In the intensity conditions, only stain intensities were varied (stain vectors fixed), whereas in the color similarity conditions only stain vectors were varied (intensities fixed). Features were extracted once for all foundation models and conditions. Visualizations of simulated tile appearances across a range of tissue regions relevant to MSI classification (including tumour epithelium, stroma, tumour-infiltrating lymphocytes, medullary morphology, mucinous differentiation, and necrosis) are provided in Supplementary Fig. D.1, illustrating the effect of each simulated staining condition and the qualitative conservation of morphological information across tissue compartments.

### Evaluation metrics

Model performance was quantified as the AUC on the reference staining condition, and robustness to staining variation as the min-max range of AUCs across the five staining conditions (reference and four simulated staining conditions). Models were considered high-performing with a reference AUC over 90% and highly robust with a min-max AUC range of less than 3%. Confidence intervals (CIs) were estimated using 1000 bootstrap resamples of the SurGen cohort. For each iteration, AUCs were computed for all five conditions, yielding a bootstrap-specific min-max range (robustness) and reference AUC (performance). To compare performance between the reference and simulated staining conditions, we calculated the difference in AUC (ΔAUC). Distribution and variability of ΔAUCs were summarized using median, quantile, and minimum statistics. Further, staining conditions were ranked by AUC for each model, and we recorded how many models achieved their highest AUC under each condition. For visualization purposes, staining characteristics were summarized using stain intensity, stain similarity as the angle between H&E vectors in OD space, and stain hue quantified as the hue angle ($h^{{}^{\circ}}=\arctan2\left(b^{*},a^{*}\right)$) in CIELab space.

### GPU usage and code availability

All code was implemented in Python, model training and inference was done with PyTorch. Training and inference were performed on NVIDIA H200 GPUs, totaling 444 GPU hours. Energy usage was measured via nvidia-smi and totaled 208.45 kWh (Supplementary Table E.1). Code for stain vector and intensity extraction, model training, inference, analysis, and visualization is publicly available on GitHub^o^. We further make PLISM and SurGen stain vectors and intensities, all 300 trained models and experiment results publicly available^p^.

## Results

### PLISM-derived staining references

**Fig. 2:**
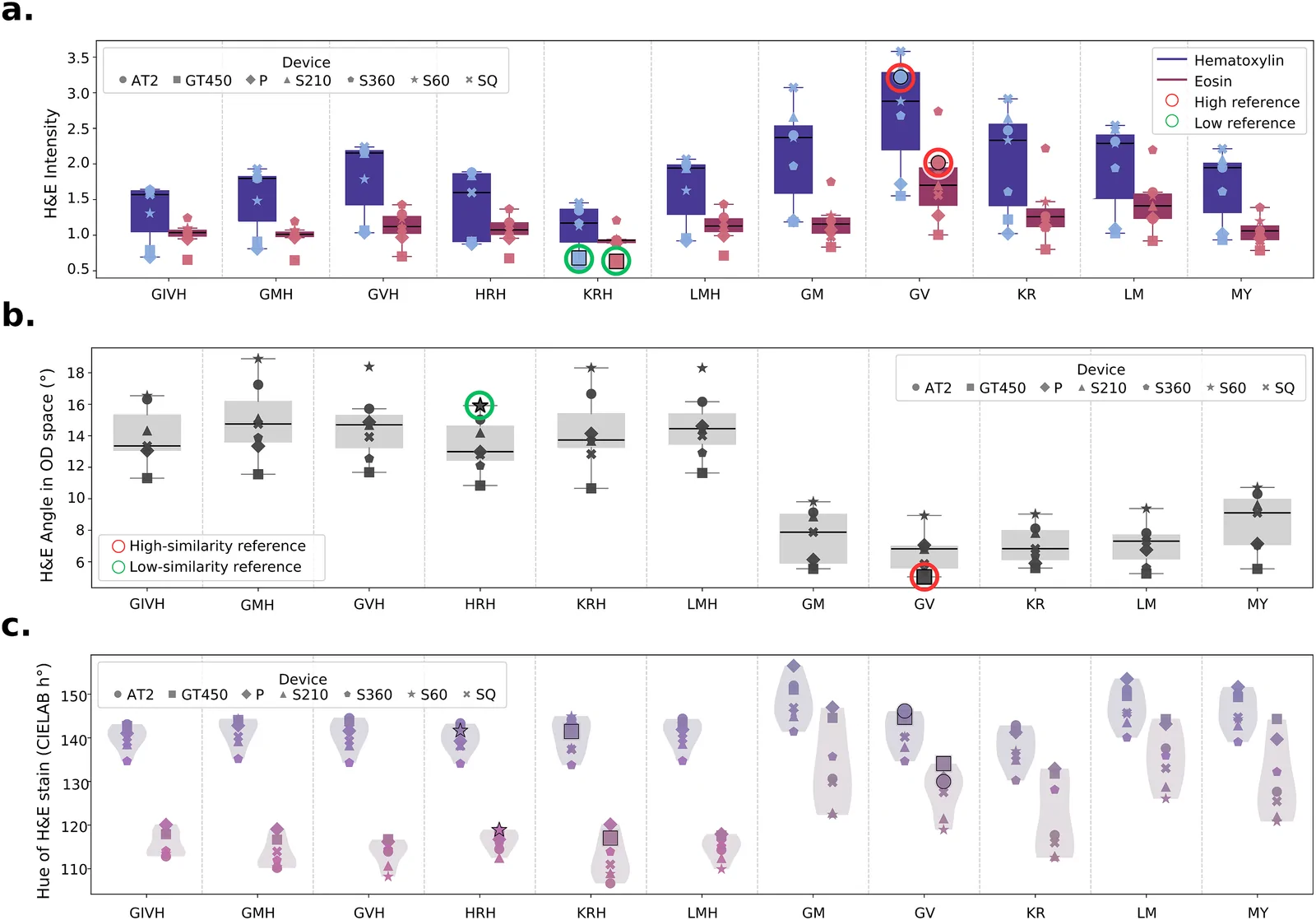
Staining characteristics of PLISM staining condition-device combinations. (a) Intensity of hematoxylin and eosin, b) angle between H&E stain vector in OD space, c) distribution of H&E hues, measured as hue h° in CIELab space; left violin: hematoxylin, right violin: eosin. Marker colors correspond to RGB stain colors. The selected reference conditions (low and high intensity; low and high H&E color similarity) are circled in red and green, respectively, and highlighted with a black frame. For staining condition and device abbreviations, please refer to Supplementary Tables A.1 and A.2. (For interpretation of the references to color in this figure legend, the reader is referred to the web version of this article.)

To establish a reference library for realistic H&E staining changes, we first characterized the H&E intensities and stain vectors for each unique PLISM stain-device condition. Consistent with the PLISM study design, conditions intended to be more distinct in H&E color (ending with “H”) showed larger angles between H&E stain vectors compared to similar H&E color conditions (Fig. 2a+b). This pattern was also reflected in H&E hues, which displayed greater separation for distinct stain color conditions (Fig. 2c). Furthermore, we note that PLISM conditions optimized for distinct H&E colors exhibited lower H&E intensities compared to higher similarity conditions (median H: 1.58 vs. 2.10; median E: 1.04 vs. 1.35). This difference did not map consistently onto a single recorded slide preparation parameter (e.g., hematoxylin formulation, exposure duration, or number of dehydration steps), suggesting a multifactorial relationship. H&E stain color similarity varied more across staining conditions than across WSI scanners (mean standard deviation (std) across devices = 1.9° vs. across stainings = 3.7°), whereas H&E intensities showed slightly more variation across scanners (Hematoxylin: mean std. across devices = 0.56 vs. across stainings = 0.47; Eosin: mean std. across devices = 0.28 vs. across stainings = 0.25).

### H&E staining variability in the SurGen dataset

**Fig. 3:**
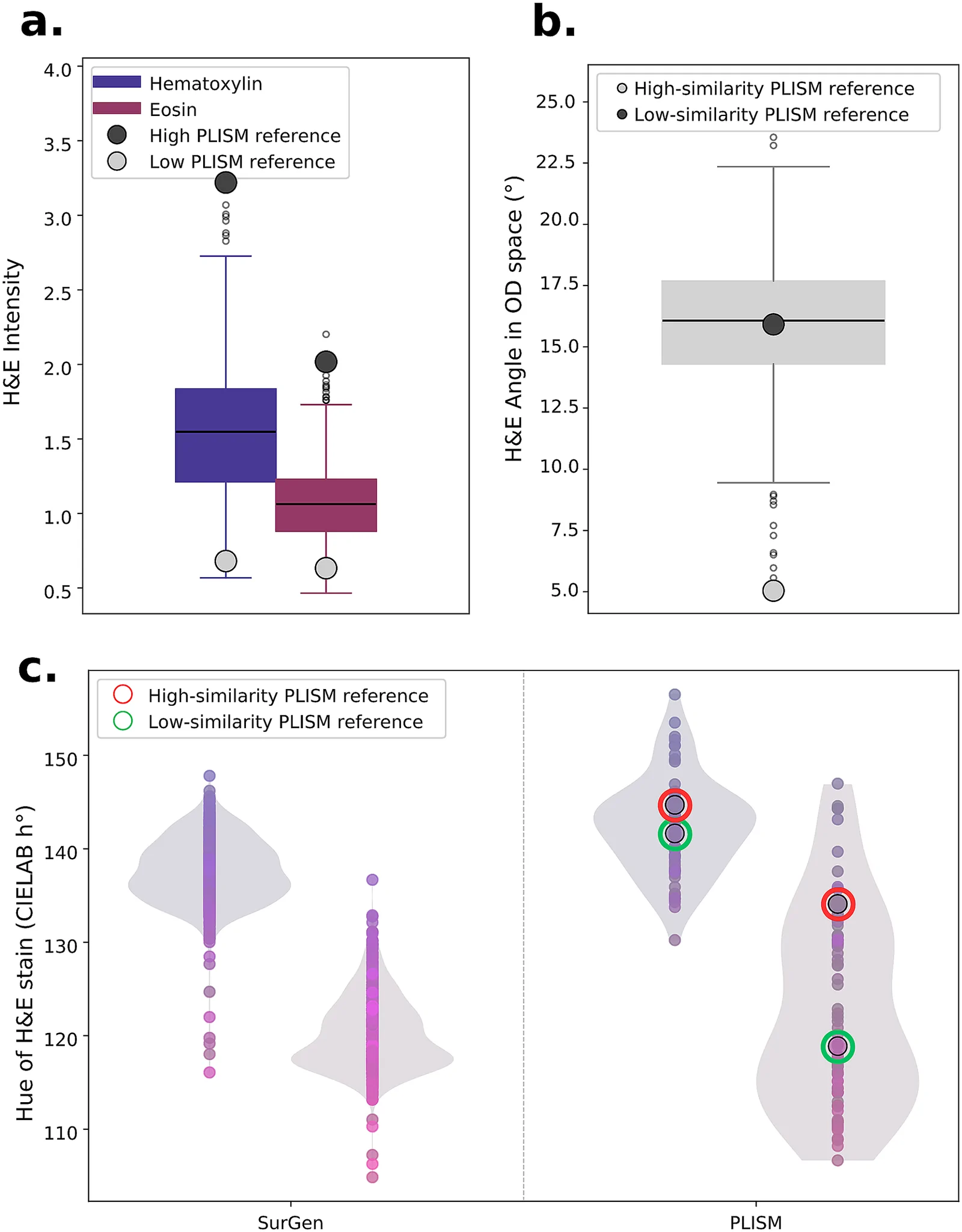
Staining characteristics of SurGen WSIs. (a) Intensity of hematoxylin and eosin, (b) angle between H&E stain vector in OD space, (c) distribution of H&E hues, measured as hue h° in CIELab space; left violin: hematoxylin, right violin: eosin. Marker colors correspond to RGB stain colors; low and high color similarity PLISM references are circled in green and red, respectively. (For interpretation of the references to color in this figure legend, the reader is referred to the web version of this article.)

Analysis of SurGen slide-level H&E intensities and stain vectors revealed substantial intrinsic variability, encompassing, and in some cases, exceeding the selected PLISM reference conditions (Fig. 3). H&E intensities ranged from 0.57 to 3.10 and 0.46 to 2.20, slightly extending beyond the low (H = 0.68, E = 0.63) and high (H = 3.22, E = 2.02) PLISM reference intensities. H&E hues also marginally extended the PLISM reference range. H&E stain color similarity, measured as the angle between H&E vectors, spanned 5.1°–23.6°, covering the PLISM “high-similarity” reference (5.0°) and surpassing the “low-similarity” reference (15.9°).

### Robustness–performance relationship in MSI classification

**Fig. 4:**
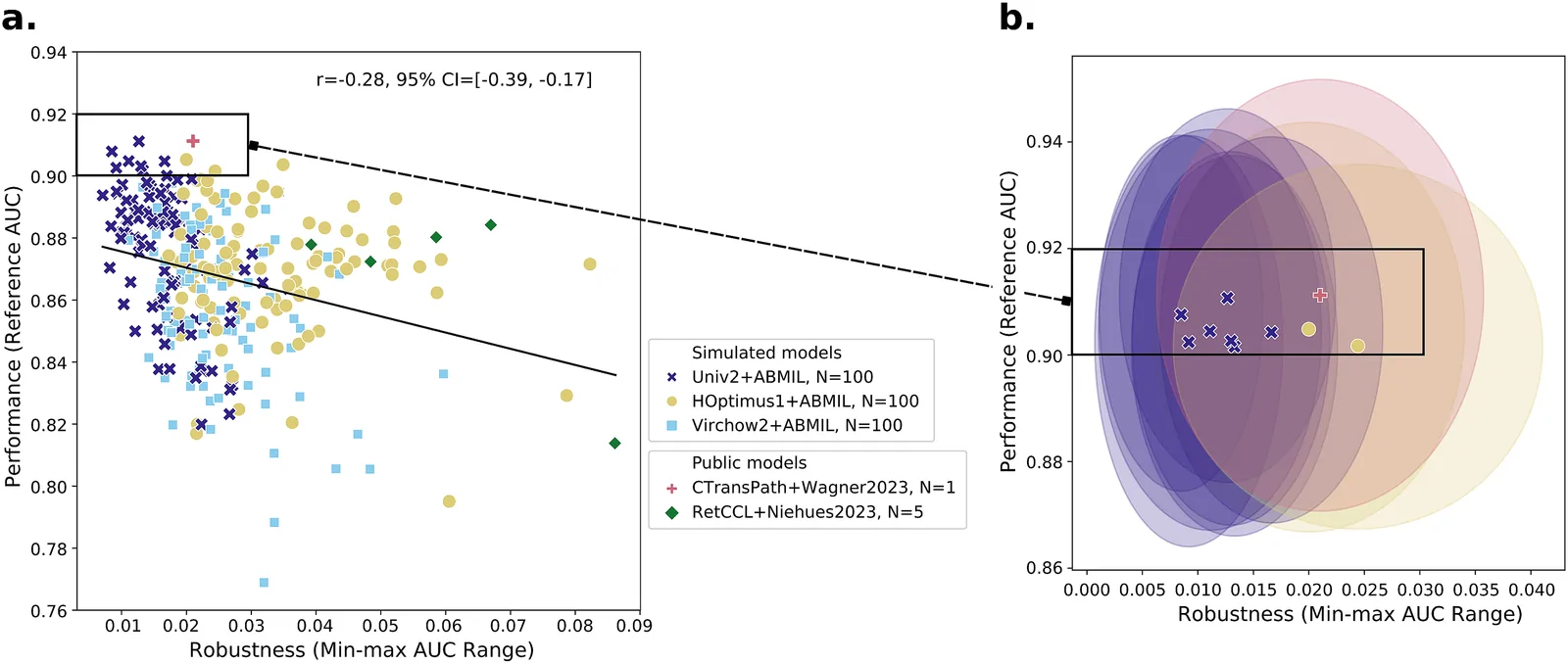
Performance-robustness relationship across MSI classification models: (a) Performance (AUC of reference condition) vs. robustness (min-max AUC across all stain conditions) for all evaluated models (*n* = 306). We report Pearson correlation between performance and robustness with 95% CIs. (b) Top models (Performance >0.90, Robustness <0.03; *n* = 10). Dots indicate bootstrapped means (*n* = 1000 iterations); ellipses represent 95% CIs for both performance and robustness.

To examine how model performance relates to robustness under staining simulations, we evaluated model performance on the SurGen dataset under the original and four simulated H&E staining conditions for the 306 MSI classification models. Across all models, reference performance ranged from AUC 0.769 to 0.911, robustness to staining variation ranged from 0.007 to 0.079 and exhibited a weak inverse association (*r* = −0.28, 95% CI = [−0.39, −0.17], *n* = 306), indicating that higher reference AUC does not necessarily imply greater robustness to staining variability (Fig. 4a). However, this varied depending on the foundation model. A moderate negative correlation was observed for UNI2-h + ABMIL (*r* = −0.51, 95% CI = [−0.63, −0.39], *n* = 100) and Virchow2+ ABMIL (*r* = −0.36, 95% CI = [−0.52, −0.19], n = 100), whereas no significant association was observed for H-Optimus-1+ ABMIL (*r* = −0.14, 95% CI = [−0.36, 0.10], *n* = 100) and the RetCCL+Niehues2023 models (*r* = −0.75, no 95% CI due to low sample size, *n* = 5). Reference AUC distributions across foundation models are summarized in Supplementary Material F, showing differences in median performance (median AUC UNI2-h: 0.881, H-Optimus-1: 0.865, Virchow2: 0.856, across *N* = 100 models), but substantial overlap in interquartile ranges (UNI2-h: [0.860, 0.892], H-Optimus-1: [0.848, 0.878], Virchow2: [0.838, 0.873]).

Considering only the high-performing, highly robust models (*n* = 10), predominantly UNI2-h + ABMIL models, alongside the CTransPath+Wagner2023 model and two H-Optimus-1+ ABMIL models remained (Fig. 4b). Among these, reference performance was very similar: the top-performing CTransPath+Wagner2023 model achieved an AUC of 0.911, closely followed by UNI2-h + ABMIL models (0.902–0.911), and slightly lower-performing H-Optimus-1+ ABMIL models (0.902–0.905). In contrast, robustness differed more clearly across models: UNI2-h + ABMIL models showed consistently smaller robustness ranges (0.009–0.013) than H-Optimus-1+ ABMIL (0.020–0.024) and CTransPath+Wagner2023 (0.021). Bootstrap analysis reinforced this separation: whereas 95% CIs for reference AUC largely overlapped across top-performing models (Fig. 4b, CTransPath+Wagner2023: [0.869,0.950]; best UNI2-h + ABMIL: [0.875,0.944]; best H-Optimus-1+ ABMIL: [0.865,0.941]), robustness CIs were narrower and more discriminative. Median robustness CI width was smallest for UNI2-h + ABMIL (0.019), compared with H-Optimus-1+ ABMIL (0.031) and CTransPath+Wagner2023 (0.030), indicating greater stability.

### Impact of staining conditions on model performance

Finally, we assessed how each of the four simulated staining conditions influenced model performance compared to the reference. Across all models (*n* = 306), the high intensity condition most frequently yielded the highest AUC (127 models), followed by the reference condition (65 models) and the low H&E color similarity condition (51 models) (Table 1). At the same time, each condition was associated with performance decreases in subsets of models, with worst-case scenarios of more than 3% performance decrease for any condition (Table 1). Restricting to high-performing, robust models (*n* = 10), high intensity remained the most favorable condition (8/10 models, median ΔAUC +0.50%, Table 1, Supplementary Fig. G.1a), achieving the overall highest AUCs on the SurGen dataset for the CTransPath+Wagner2023 model and a single H-Optimus-1+ ABMIL model (both: 0.920), exceeding their respective reference-condition performance (CTransPath+Wagner2023: 0.911, H-Optimus-1+ ABMIL: 0.904). Simultaneously, low intensity and high H&E color similarity led to modest median decreases (−0.46% and −0.32%, Table 1, Supplementary Fig. G.1b). Worst-case AUC drops also followed a condition-dependent pattern: decreases were minimal (<0.4%) for high intensity and distinct H&E color conditions, but larger (>1%) for low intensity and similar H&E color conditions.

**Table 1:** Effects of simulated H&E staining conditions on all models (*n* = 306) and top-performing, robust models (*n* = 10). For each condition, the table reports the number of models for which it performed best, the median ΔAUC relative to reference with 95% CI, and the worst-case AUC decrease compared to reference.

Staining condition	Best model count	Median ΔAUC [95% CI]	Worst-case AUC decrease
**All models(*****n*** **=** **306)**			
Reference	65	N/A	N/A
Low intensity	30	−0.50% [−1.13%, −0.05%]	−4.50%
High intensity	127	0.12% [−0.65%, 0.73%]	−4.36%
Low H&E color similarity	51	−0.07% [−0.52%, 0.34%]	−3.17%
High H&E color similarity	33	−0.57% [−1.57%, 0.11%]	−7.78%
**Top models (*n*** **=** **10)**			
Reference	0	N/A	N/A
Low intensity	0	−0.46% [−0.63%, −0.07%]	−1.08%
High intensity	8	0.50% [0.36%, 0.73%]	−0.37%
Low H&E color similarity	2	0.24% [0.05%, 0.39%]	−0.20%
High H&E color similarity	0	−0.32% [−0.62%, −0.02%]	−1.87%

## Discussion

This study provides an evaluation protocol to: (1) quantify the robustness of CPath models to H&E staining variability and to (2) systematically assess how staining conditions affect model performance. Across a large population of MSI classification models, we observed substantial heterogeneity in robustness against staining variability, with only a weak inverse relationship between performance and robustness. This suggests that higher predictive performance does not guarantee stability under staining shifts, supporting the notion that robustness to staining variation is a partially independent property of trained models^4^, ^5^. Notably, we found that this pattern persists even in foundation-model-based pipelines, and becomes particularly evident when evaluating many downstream predictors under simulated staining conditions, rather than focusing on a single best-performing model. Unexpectedly, UNI2-h and H-Optimus-1 foundation models were found to be more robust than Virchow2, contrasting prior reports^20^, ^21^. Furthermore, the Wagner et al.^43^ model, though built on the less robust CTransPath foundation model^44^, ranked among the top three in both performance and robustness to staining variation, suggesting that aggregator training can mitigate foundation model sensitivity to staining variability. Overall, we found that high-performing, robust models exist and can be identified by considering both metrics jointly. Systematic trends in model performance emerged across staining conditions. Across all models, high intensity and low H&E color similarity conditions more frequently yielded top performance, whereas low intensity and high H&E color similarity tended to produce slightly negative shifts relative to the reference. However, these effects were not universal, as all conditions produced the best AUCs for subsets of models. Considering only high-performing, robust models, the same patterns were more pronounced: high intensity and low H&E color similarity most often corresponded to the highest AUCs, whereas low intensity and high H&E color similarity produced modest decreases. Nonetheless, individual models continued to deviate from these trends, indicating that the effect of staining conditions remains model-dependent and should be assessed individually. Further, our analysis adds a key piece in understanding the origin of staining variability: in the PLISM dataset, H&E stain color similarity varied more across staining protocols than across scanners, whereas H&E intensities showed slightly more variation across scanners than protocols, suggesting that color and intensity capture partially distinct sources of variability attributable to protocol and scanner respectively. As staining protocols can be adjusted within existing workflows without significant additional costs, in contrast to scanner infrastructure, they represent the more practical starting point for ensuring staining conditions remain within a desired operational range. In practice, we recommend first characterizing the staining properties of routine slides with available scanners, then identifying protocol adjustments that keep staining conditions within this range, supporting consistent WSI quality and reliable model performance.

Prior work on evaluating CPath model robustness to staining variation has focused on using image-based references^25^, ^26^, generative^28^ or physically repeated staining protocols^29^, ^30^, ^31^. Although establishing the clinical relevance of staining variability, they do not anchor perturbations to quantifiable stain intensities or similarity measures within a defined reference space. Consequently, attribution of performance changes to specific staining properties remains challenging. In contrast, our protocol applies deterministic, reference-based staining simulations that explicitly control stain intensity and similarity, enabling systematic testing of trained models under defined staining conditions. This enables interpretable assessment of robustness to staining variation and provides a structured, reference-based evaluation protocol that complements existing training- and evaluation-focused approaches.

Our study has several limitations. First, the empirical findings presented here are specific to CRC, MSI prediction, the studied classification models and staining condition set. Therefore, extension to other cancer types, diagnostic tasks, stain ranges, and model families requires further validation. Second, the PLISM reference library captures only part of the staining variability observed in clinical slides, with the SurGen cohort often exceeding its range for H&E stain angles, indicating that capturing additional stain preparation protocols and scanner combinations would be beneficial. Whereas larger reference sets with pre-extracted stains exist^13^, they do not allow verification of color correctness, lack intensity properties as well as traceable diagnostic conditions. PLISM, in contrast, provides known staining properties and scanner origins across a broad range of tissue types. Third, our protocol deliberately isolates H&E staining color and intensity variation as a well-defined and quantifiable subset of the broader variability space encountered in routine histopathology. Real-world H&E appearance is additionally shaped by pre-analytical factors, such as fixation time, section thickness, differentiation, and dehydration, processing artifacts such as tissue folds or air bubbles as well as scanner-specific effects on sharpness, compression, and focus, none of which are modeled here. The protocol should therefore be understood as a focused stress-test of induced staining variation, with prior work showing that staining is among the strongest factors influencing model performance^24^, ^25^, ^26^, ^27^. Fourth, robustness is assessed using AUC, which captures rank-based discriminative performance but does not fully reflect clinical stability. A model may preserve its AUC across staining conditions while showing relevant shifts in score calibration, which are important additional dimensions of robustness for clinical deployment. Fifth, slide-level stain property extraction and simulation rely on a stain decomposition approach calibrated for standard tissue regions, where H&E are present at typical concentrations. Tissue components such as blood, coagulative necrosis, or heavily pigmented areas deviate from this assumption and are therefore not accurately captured, both in terms of stain property estimation, where their inclusion can hamper the estimated stain vectors, and simulation, where the appearance of these components may not be fully realistic under staining variation. We mitigated this risk in stain property estimation by employing quality criteria and semi-automated outlier detection, with a bootstrapping sensitivity analysis confirming stable estimates from 10 tiles within the current approach (Supplementary Material C). At the same time, the tile selection remains a key area of improvement, particularly to enable its full automization. Future work should therefore explore more robust tile selection solutions such as dedicated artifact segmentation models, or extend the decomposition algorithm to better model staining properties across all tissue components. Lastly, only four discrete staining conditions were tested, limiting exploration of continuous or non-linear effects.

## Conclusion

We present a protocol for systematically quantifying the robustness to H&E staining variation of CPath models, revealing how specific H&E staining properties impact performance across a broad set of MSI classification models. Our analysis suggests that robustness to H&E staining variation is a model-specific property, only partially correlating with overall predictive performance, and highlights the interplay between staining protocols and scanner choice in shaping digital appearance. Staining conditions produced trends in model performance: in our experiments, high intensity was associated with slightly better performance, whereas low intensity or highly similar H&E colors tended to cause modest decreases. However, this was not universal, with subsets of models showing reverse effects, highlighting the need for model-specific evaluation. These results demonstrate that the proposed protocol provides a structured and reproducible framework for evaluating model robustness to H&E staining variation, and revealed both general trends and model-specific sensitivities of MSI classification models in CRC, thereby providing actionable guidance for robust model deployment.

Our findings highlight actionable directions for both clinical implementers and algorithm developers in advancing stain-robust CPath. For clinical implementers, we recommend the following practical steps: first, characterize the staining properties of routine WSIs, including H&E intensity and stain vectors, across a representative set of local slides, covering different staining batches, reagent lots, and scanners in use. This establishes the lab’s operational staining range, defined by the distribution of stain vectors and intensities observed under routine conditions, which serves as a reference for ongoing quality control. Systematic deviations from this reference range over time can then be interpreted as temporal “concept drift” in staining characteristics, for example, due to changes in protocols, reagents, or scanner conditions, and used to flag outlier cases for targeted re-staining or re-scanning. To improve deployment reliability of CPath models, their performance should be validated against the locally observed staining range, either using real slides spanning that range or by simulating staining conditions at the boundaries of that range using protocols like ours. This helps ensure that the model performs reliably under the staining conditions it will encounter in routine use, and provides a basis for defining acceptable operating boundaries for clinical deployment. For algorithm developers, creating reference libraries that capture realistic, traceable, and institution-relevant staining conditions is critical. Public datasets often lack intensity information or include unrealistic color variations, limiting their utility for QC. Combining publicly available references with locally characterized staining properties enables defining operational ranges in which models perform reliably. Ideally, standardized reference sets could be harmonized across institutions and countries to define minimal “fit for purpose” robustness requirements for CPath models, supporting domain generalization, regulatory confidence, and trust in clinical deployment. Future work should extend robustness evaluation beyond rank-based discriminative performance to include absolute score stability, calibration shifts, and scaling drifts under staining changes. Expanding these analyses to other tasks, modalities (e.g., IHC), and systematically studying interactions between staining, scanning, and training strategies would provide a more complete understanding of model sensitivity and guide both algorithm development and clinical quality assurance, supporting reliable and generalizable deployment of CPath models.

## CRediT authorship contribution statement

**Lydia A. Schoenpflug:** Conceptualization, Data curation, Formal analysis, Investigation, Methodology, Project administration, Software, Validation, Visualization, Writing – original draft, Writing – review & editing. **Nikki J. van den Berg:** Conceptualization, Writing – review & editing. **Sonali Andani:** Conceptualization, Writing – review & editing. **Nanda Horeweg:** Conceptualization, Supervision, Writing – review & editing. **Jurriaan Barkey Wolf:** Software, Writing – review & editing. **Tjalling Bosse:** Conceptualization, Supervision, Writing – review & editing. **Viktor Koelzera:** Conceptualization, Funding acquisition, Supervision, Writing – review & editing. **Maxime W. Lafarge:** Conceptualization, Methodology, Project administration, Software, Supervision, Writing – review & editing.

## Declaration of generative AI and AI-assisted technologies in the manuscript preparation process

During the preparation of this work, the authors used ChatGPT in order to structure and polish the text. After using this tool, the authors reviewed and edited the content as needed and take full responsibility for the content of the published article.

## Funding

This research did not receive any specific grant from funding agencies in the public, commercial, or not-for-profit sectors. The work was supported by core institutional funding from the University of Basel.

## Declaration of competing interest

The authors declare the following financial interests/personal relationships which may be considered as potential competing interests: Lydia Schoenpflug reports a relationship with Indica Labs that includes: travel reimbursement. Nanda Horeweg reports a relationship with Dutch Cancer Society that includes: funding grants. Tjalling Bosse reports a relationship with Dutch Cancer Society that includes: funding grants. Tjalling Bosse reports a relationship with Hanarth Fund Foundation that includes: funding grants. Viktor Koelzer reports a relationship with F. Hoffmann-La Roche Ltd. that includes: consulting or advisory and funding grants. Viktor Koelzer reports a relationship with Image Analysis Group that includes: funding grants. Viktor Koelzer reports a relationship with Takeda that includes: consulting or advisory. Viktor Koelzer reports a relationship with Indica Labs that includes: speaking and lecture fees. Viktor Koelzer reports a relationship with Sharing Progress in Cancer Care that includes: speaking and lecture fees. Viktor Koelzer has patent Methods for Predicting Treatment Outcome and/or for Selecting a Subject Suitable for Immune Checkpoint Therapy issued to The Netherlands Cancer Institute. Viktor Koelzer has patent Improved Method for Predicting Efficacy of Cancer Treatment pending to University of Oxford. Viktor Koelzer has patent Methods for Training and Using a Machine Learning Model for Predicting a Likelihood of an Event Occurring in a Patient Having a Malignant Tumour by Computational Pathology issued to Leiden University Medical Center. Tjalling Bosse has patent Methods for Training and Using a Machine Learning Model for Predicting a Likelihood of an Event Occurring in a Patient Having a Malignant Tumour by Computational Pathology pending to Leiden University Medical Center. Nanda Horeweg has patent Methods for Training and Using a Machine Learning Model for Predicting a Likelihood of an Event Occurring in a Patient Having a Malignant Tumour by Computational Pathology pending to Leiden University Medical Center. If there are other authors, they declare that they have no known competing financial interests or personal relationships that could have appeared to influence the work reported in this article.

## Footnotes

e https://huggingface.co/datasets/owkin/plism-dataset-tiles, last accessed 04.12.2025.

f https://portal.gdc.cancer.gov/, last accessed 04.12.2025.

g https://www.cbioportal.org/, last accessed 04.12.2025.

h https://www.ebi.ac.uk/biostudies/bioimages/studies/S-BIAD1285, last accessed 04.12.2025.

i https://huggingface.co/MahmoodLab/UNI2-h, last accessed 04.12.2025.

j https://huggingface.co/bioptimus/H-Optimus-1, last accessed 04.12.2025.

k https://huggingface.co/paige-ai/Virchow2, last accessed 04.12.2025.

l CTransPath: https://huggingface.co/jamesdolezal/CTransPath; RetCCL^43^, ^45^: https://github.com/Xiyue-Wang/RetCCL; https://github.com/peng-lab/HistoBistro/tree/main/CancerCellCRCTransformer; https://github.com/KatherLab/crc-models-2022/tree/main/Quasar_models/Wang%2BattMIL/isMSIH; all last accessed 15.01.2026.

m PLISM stain references on HuggingFace: https://huggingface.co/datasets/CTPLab-DBE-UniBas/staining-robustness-evaluation/tree/main/plism-wsi_stain_references.

n SurGen stain references on HuggingFace: https://huggingface.co/datasets/CTPLab-DBE-UniBas/staining-robustness-evaluation/tree/main/surgen_stain_properties.

o GitHub Repository: https://github.com/CTPLab/staining-robustness-evaluation/tree/main.

p HuggingFace Repository: https://huggingface.co/datasets/CTPLab-DBE-UniBas/staining-robustness-evaluation/tree/main.
