# Supplementary material for: A protocol for evaluating robustness to H&E staining variation in computational pathology models
Source: J Pathol Inform. 2026 Aug 1;22:100702. doi: 10.1016/j.jpi.2026.100702 (PMC13545398; doi:10.1016/j.jpi.2026.100702)
Supplement: Supplementary file 1 [file mmc1.pdf]

Supplementary Material

A PLISM staining condition and device abbreviations

Table A.1: PLISM diagnostic stain preparation condition parameters and abbreviations.

**Hematoxylin Type (Product No.)**	**Hematoxylin Exposure (min)**	**Eosin Exposure (min)**	**Dehydration Steps**	**Abbreviation**
Gill IV (8647)	0.5	15	1	GIVH
GM (30081)	2	15	1	GMH
New Type G (30161)	5	15	4	GM
Gill V (20032)	5	15	1	GVH
Gill V (20032)	60	15	4	GV
Mayer (30002)	3	3	1	MY
Harris (20022)	2	15	1	HRH
Carrazi (30131)	5	15	1	KRH
Carrazi (30131)	60	15	4	KR
Lillie-Mayer (30072)	2	15	1	LMH
Lillie-Mayer (30072)	2	15	5	LM

Table A.2: PLISM WSI scanning device abbreviations.

**Device**	**Abbreviation**
Hamamatsu NanoZoomer-S360	S360
Hamamatsu NanoZoomer-S210	S210
Hamamatsu NanoZoomer-SQ	SQ
Hamamatsu NanoZoomer-S60	S60
Leica Aperio AT2	AT2
Leica Aperio GT450	GT450
Phillips Ultrafast Scanner	P

B Details on PLISM reference set creation

Table B.1: Tile quality criteria used for intensity and stain vector extraction in PLISM and SurGen. Sharpness is measured as the minimum variance of the Laplacian, colorfulness as the product of HSV hue variance and mean saturation.

Quality Criterion	PLISM	SurGen
Saturation	≥30th Percentile	≥0.6
Entropy	≥30th Percentile	≥6.4
Sharpness	≥20th Percentile	≥100
Fraction red/yellow pixels	≤40th Percentile	≤0.2
Fraction green pixels	≤40th Percentile	≤0.01
Colorfulness	≥30th Percentile	≥0.008

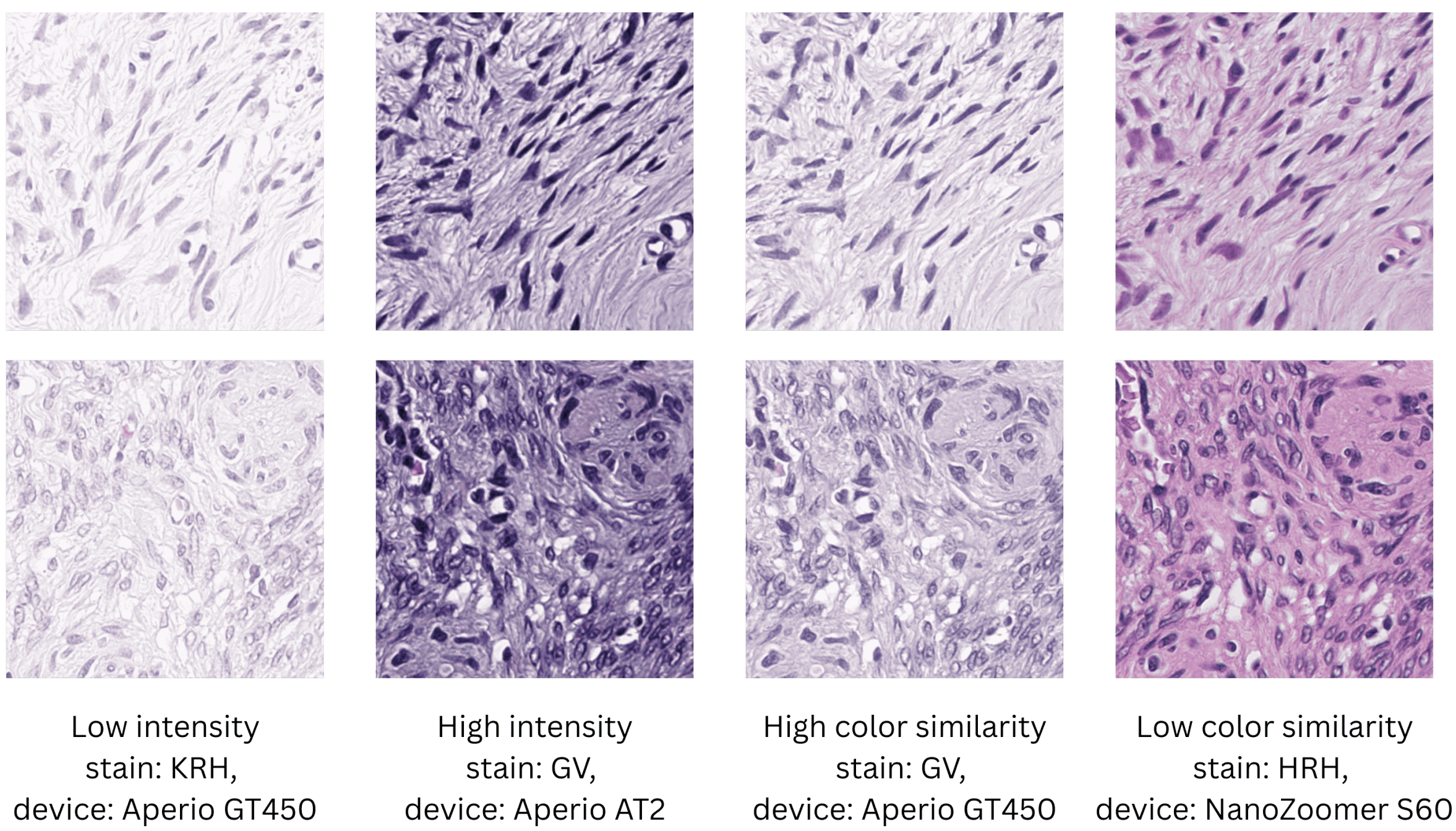

Figure B.1: Sample tiles from the chosen PLISM stain-device conditions. Low intensity: KRH, device: Aperio GT450; High intensity: GV, device: Aperio AT2; High color similarity: GV, device: Aperio GT450; Low color similarity: HRH, device: NanoZoomer S60.

Table B.2: Reference staining conditions used for model evaluation.

Reference name	Staining condition	Intensity	Stain Color	
			H	E
Low intensity	PLISM, stain: KRH, device: Aperio GT450	H: 0.68, E: 0.63	N/A	
High intensity	PLISM, stain: GV, device: Aperio AT2	H: 3.22, E: 2.02	N/A	
High color similarity	PLISM, stain: GV, device: Aperio GT450	N/A		
Low color similarity	PLISM, stain: HRH, device: NanoZoomer S60	N/A		

C Sensitivity analysis of slide-level stain property estimation

To assess whether sampling 10 quality-controlled tiles per WSI produces stable slide-level stain estimates, we conducted a bootstrapping sensitivity analysis. For each WSI, n=50 tiles were sampled using the same quality criteria as the main protocol. Stain vectors and intensities estimated from all 50 tiles served as a per-slide reference. Subsets of n=10 tiles were then repeatedly drawn with replacement (1000 iterations per WSI), and stain vectors and intensities were estimated for each subset. Sampling stability was quantified as the standard deviation of the angular and absolute intensity difference to the 50-tile reference across iterations, summarised across all WSIs (Table C.1).

Table C.1: Bootstrap sensitivity analysis of stain vector and intensity estimation (n=1000 iterations per WSI, subsets of n=10 tiles drawn from n=50 quality-controlled tiles). Values represent the standard deviation of the difference to the 50-tile reference, summarised across all WSIs.

	H angle (°)	E angle (°)	H intensity	E intensity
Median std	0.341	0.485	0.045	0.034
IQR	[0.259, 0.454]	[0.352, 0.641]	[0.034, 0.060]	[0.026, 0.047]

Sampling variability was low across the dataset. The median standard deviation of the hematoxylin and eosin stain angle differences were 0.34° (IQR: 0.26°–0.45°) and 0.48° (IQR: 0.35°–0.64°) respectively, with intensity standard deviations of 0.045 (IQR: 0.034–0.060) and 0.034 (IQR: 0.026–0.047). These results support the stability of 10-tile sampling for slide-level stain characterisation under standard tissue compositions.

D Visual assessment of simulated staining conditions across tissue compartments

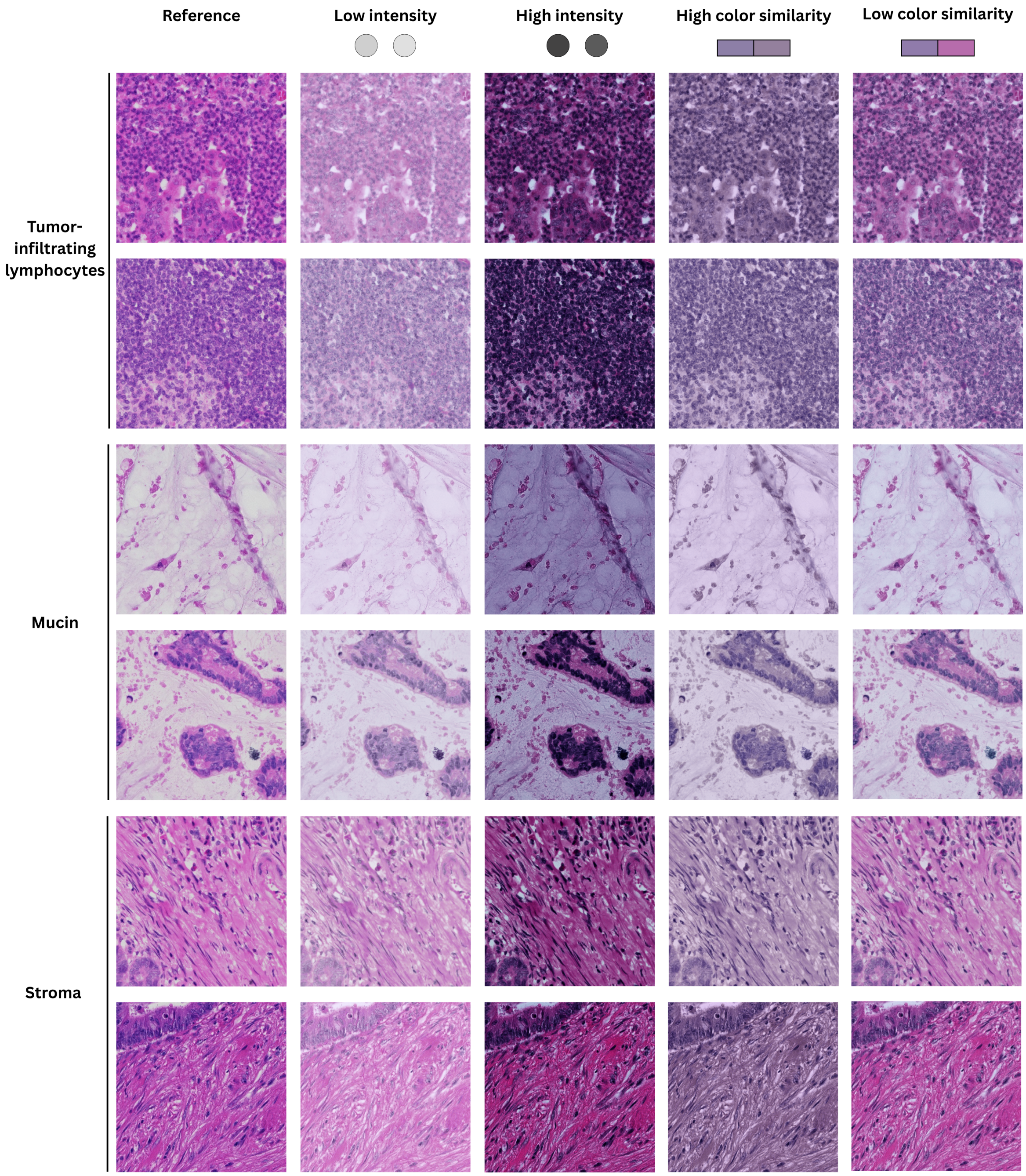

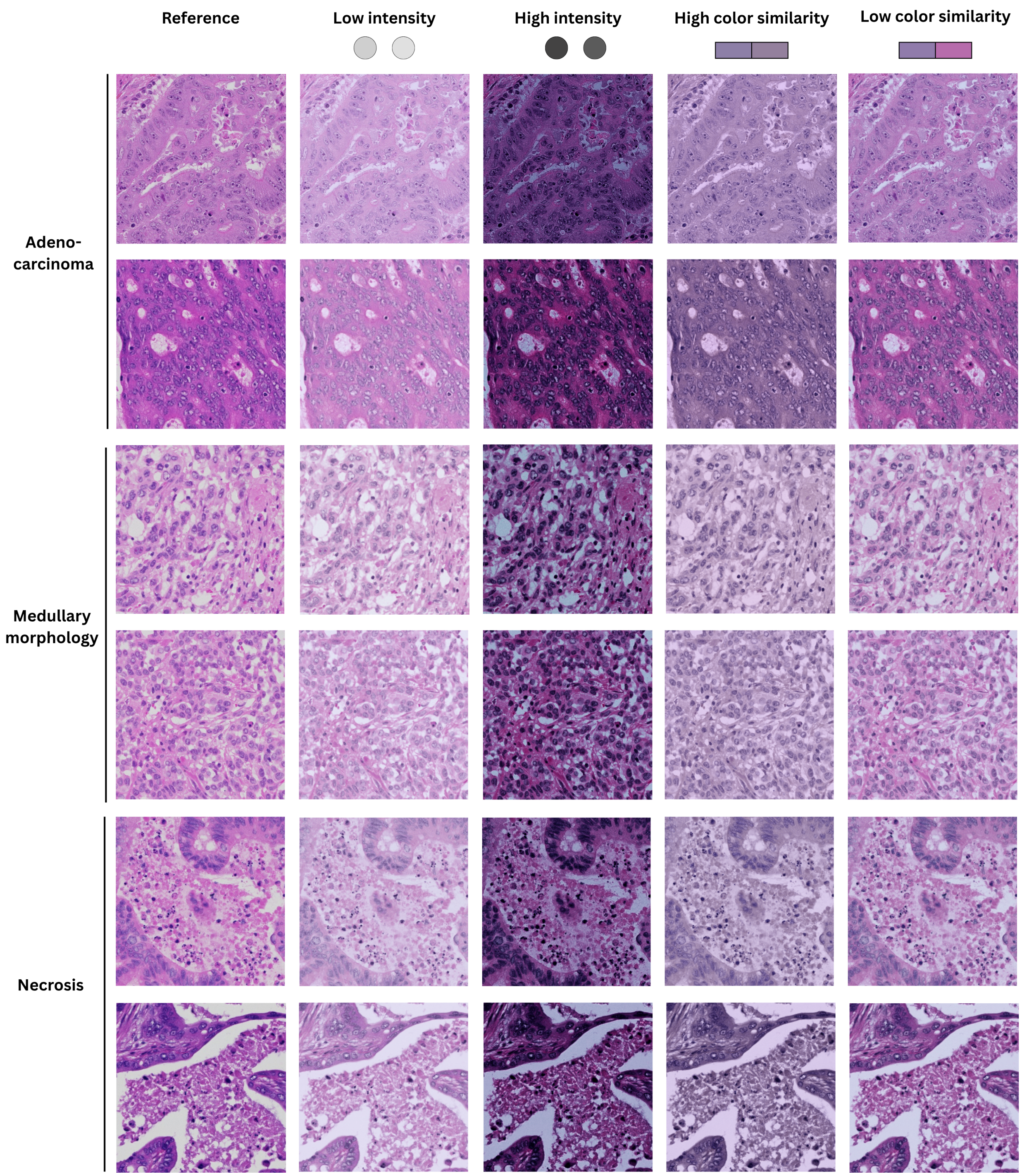
Reference
Low intensity
High intensity
High color similarity
Low color similarity
Adeno-
carcinoma
Medullary
morphology
Necrosis

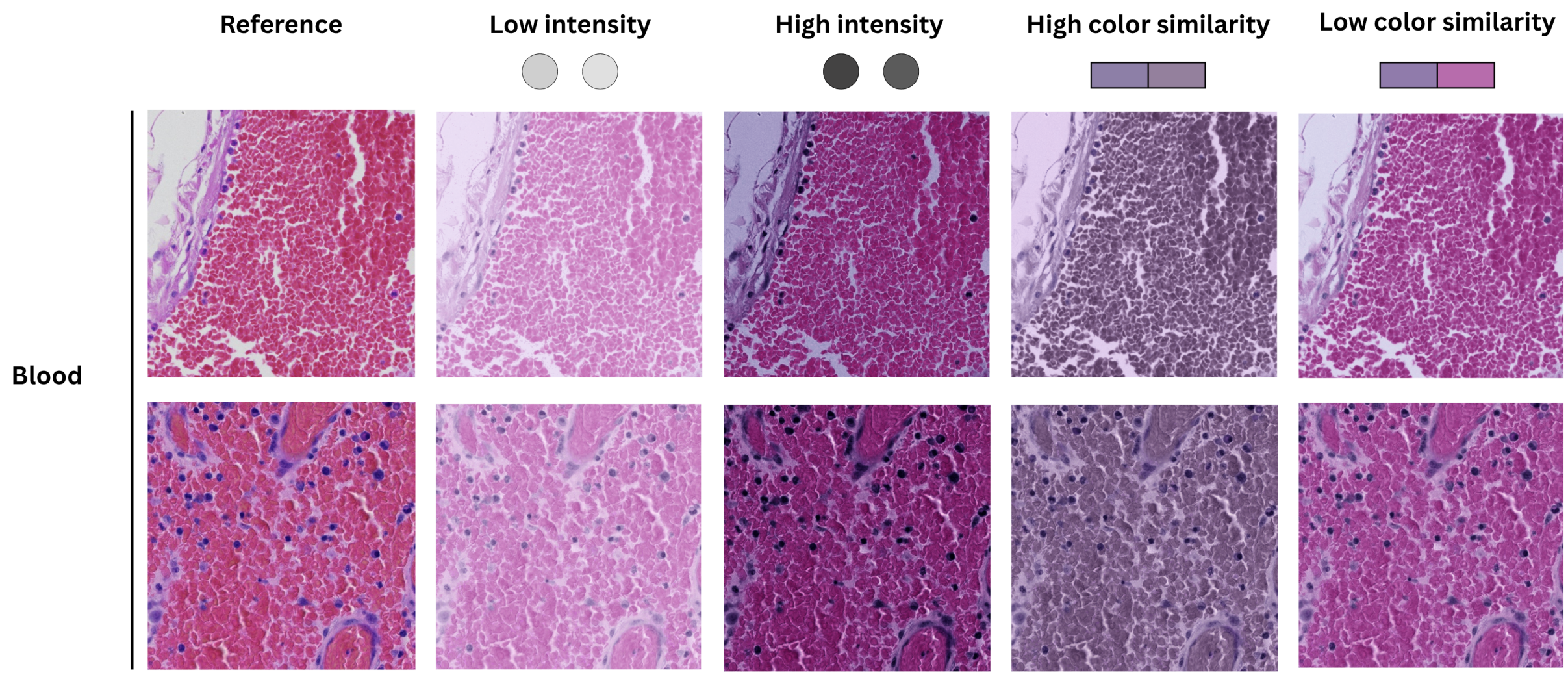

Figure D.1: Representative image tiles showing tumour-infiltrating lymphocytes (TILs), mucinous differentiation, stromal patterns, adenocarcinoma, medullary morphology, necrosis and blood under the reference staining protocol and all four simulated staining conditions. Blood-containing regions illustrate a known limitation of the stain decomposition model, where simulated colors may deviate from realistic visual appearances. For all other tissue compartments, the simulated changes appear visually consistent and biologically plausible, reflecting protocol-level staining variation propagated according to each region's staining characteristics.

E Computational costs of the study

Table E.1: GPU runtime and energy consumption per experimental step using NVIDIA H200 GPUs.

**Project Step**	**Model/ Method**	**WSIs**	**Total Runtime**	**Avg. Time per Unit**	**Energy (kWh)**
Tissue Detection	–	1337	01:03 h	2.8 s / WSI	0.12
Feature Extraction (x5 staining conditions)	UNI2-h	1337	111:30 h	1:00 min / WSI	68.89
	H-Optimus-1	1337	143:00 h	1:17 min / WSI	72.12
	Virchow2	1337	103:01 h	0:56 min / WSI	54.96
	CTransPath	1337	15:35 h	0:15 min / WSI	2.69
	RetCCL	1337	15:20 h	0:15 min / WSI	1.55
Model Training	ABMIL (300x)	599	29:09 h	05:49 min / model	4.81
Model Inference (x5 staining conditions)	ABMIL (300x)	738	25:20 h	01:00 min / model	3.30
	Niehues2023 (5x)	738	00:15 h	00:34 min / model	0.005
	Wagner2023 (1x)	738	00:02 h	00:21 min / model	0.001
**Total**	–	–	**444:24 h**	–	**208.45**

F Foundation model performance across staining conditions

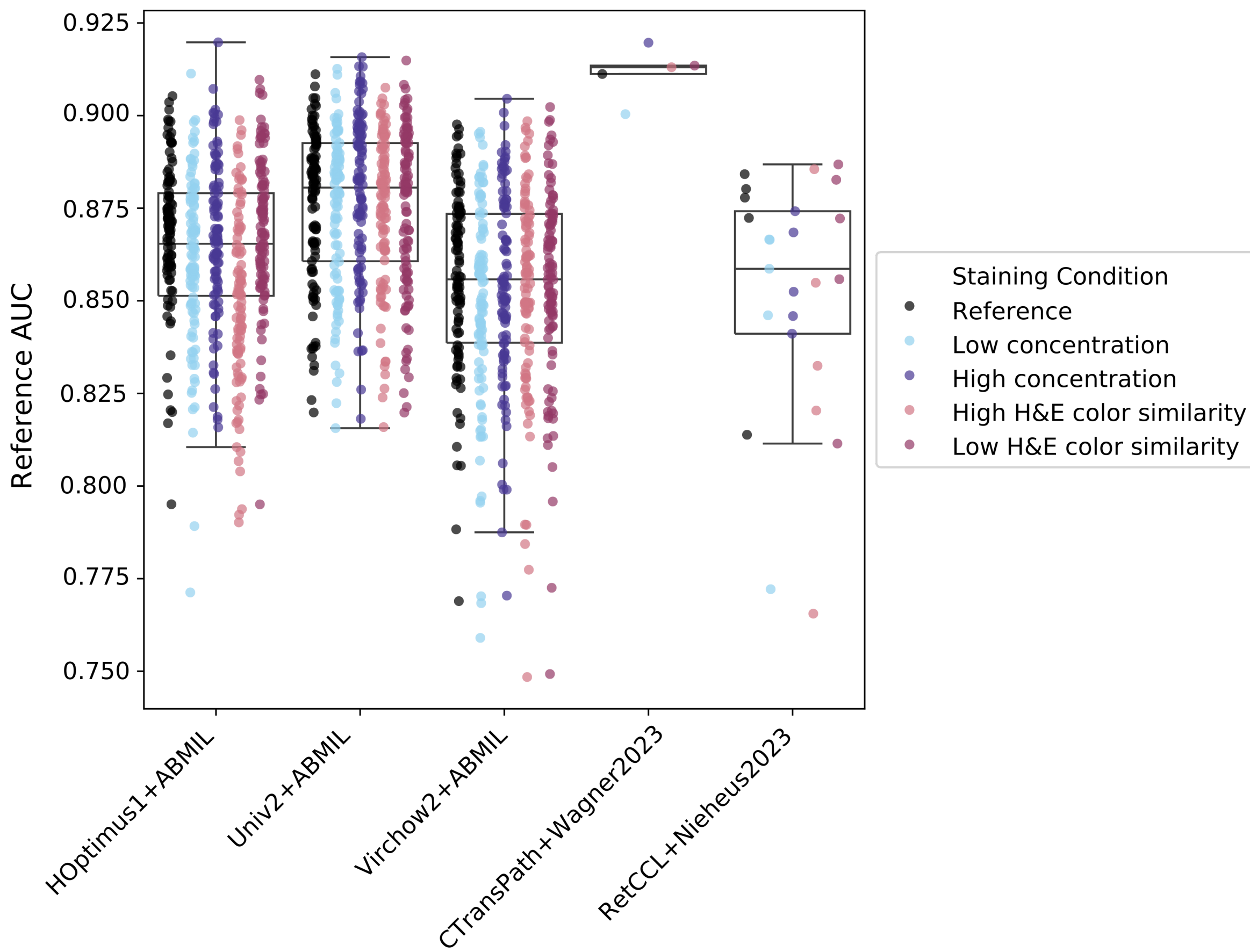

Figure F.1: Comparison of foundation model performance (bootstrapped mean reference AUC) across all staining conditions. Boxes show the median and IQR, whiskers represent 1.5x IQR.

To explore whether different foundation models differ in overall predictive performance, we compared the AUC distributions across all staining conditions. Boxplots (Figure F.1) show that median performance varied modestly between models, with CTransPath+Wagner2023 achieving the highest median AUC (0.913), followed by UNI2-h+ABMIL (0.881), H-Optimus-1+ABMIL (0.865), RetCCL+Niehues2023 (0.859), and Virchow2+ABMIL (0.856). A Kruskal-Wallis test confirmed significant differences among models (H=208.861, p¡0.001). Post-hoc pairwise Dunn's tests with Bonferroni correction indicated that most differences were statistically significant; the only non-significant comparisons were CTransPath+Wagner2023 vs. UNI2-h+ABMIL, RetCCL+Niehues2023 vs. Virchow2+ABMIL, and UNI2-h+ABMIL vs. H-Optimus-1+ABMIL. Overall, although certain foundation models showed slightly higher median performance, the interquartile ranges strongly overlapped across models (UNI2-h+ABMIL: [0.860,0.892], Virchow2+ABMIL: [0.838, 0.873], H-Optimus-1+ABMIL: [0.848,

0.878]). We only considered the three foundation models UNI2-h, H-Optimus-1 and Virchow2 with n=100 MSI classification models each, to ensure a fair statistical comparison. These results support the focus on robustness to staining variability, rather than solely considering performance, as the primary criterion for selecting a foundation model in downstream CPath applications.

G Impact of staining conditions on model performance

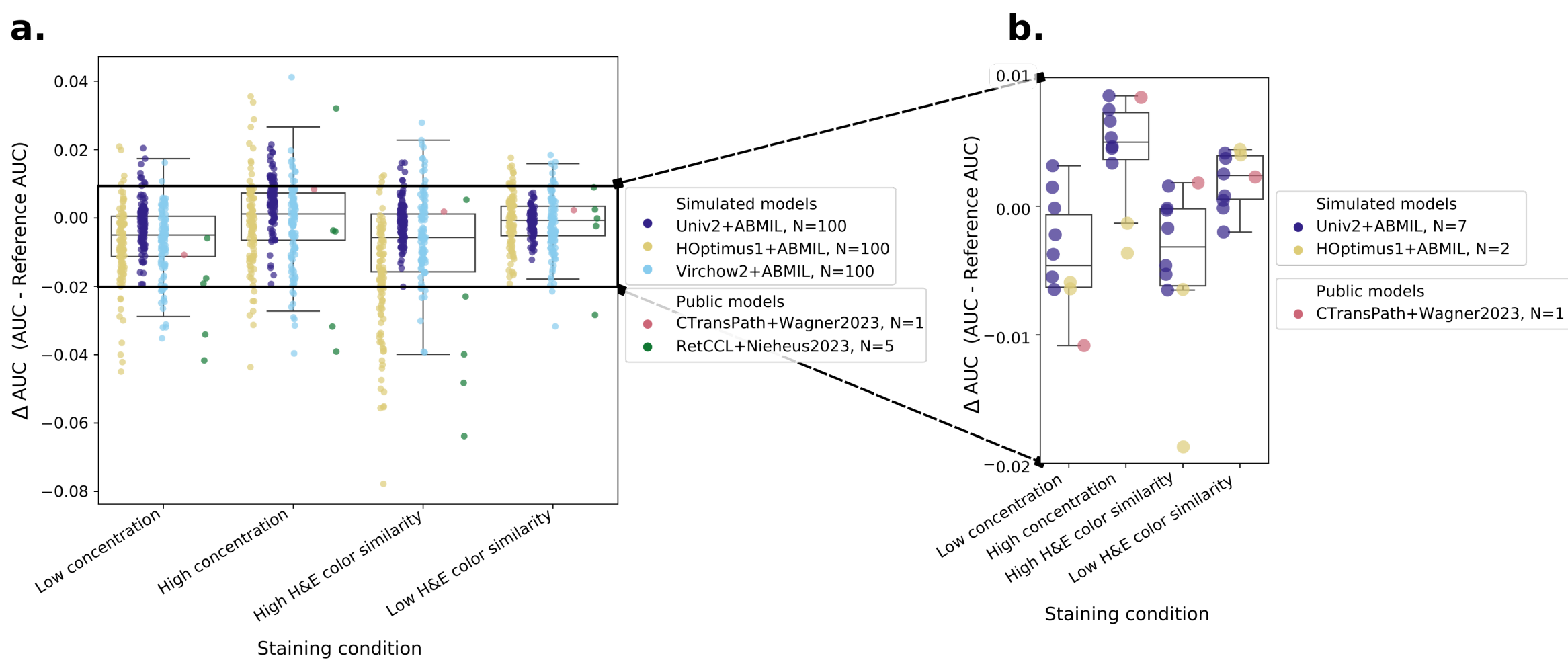

Figure G.1: Comparison of staining condition performance to reference (ΔAUC = bootstrapped mean AUC staining condition - bootstrapped mean AUC reference condition) across a. all models (n=306), b. subset of top-performing, most robust models (n=10). Boxes show the median and IQR, whiskers represent 1.5x IQR.

We assessed how each of the four simulated staining conditions influenced model performance compared to the reference. Wilcoxon tests revealed significant differences in model performance (Figure G.1) for both intensity and the high color similarity conditions, but with very modest changes in median ΔAUC (high intensity:+0.12%, low intensity: -0.50%, high color similarity: -0.57%, all $p<0.05$). The low color similarity staining condition did not differ significantly from the reference (-0.07% ΔAUC, $p>0.05$). Overall, IQRs overlapped substantially across conditions, indicating limited systematic differences in performance between staining conditions, with slight trends towards maintained performance compared to the reference for high intensity and low color similarity and lower performance for low intensity and high color similarity. These trends got further exemplified in the top-performing, most robust models, with increased performance for high intensity and low H&E color similarity (median ΔAUC = 0.50% and 0.24%, $p<0.05$ in Wilcoxon test), and lower performance for low intensity and high H&E color similarity (median ΔAUC = -0.46% and -0.32%, $p<0.05$ in Wilcoxon test).
